# Increased ratio of dietary carbohydrate to protein shifts the focus of metabolic signaling from skeletal muscle to adipose

**DOI:** 10.1186/1743-7075-8-13

**Published:** 2011-03-04

**Authors:** Suzanne Devkota, Donald K Layman

**Affiliations:** 1 Committee on Molecular Metabolism and Nutrition, The University of Chicago, Chicago, IL 60637, USA; 2 Department of Medicine, The University of Chicago, Chicago, IL 60637, USA; 3 Division of Nutritional Sciences, University of Illinois, Urbana, IL 61801, USA; 4 Department of Food Science & Human Nutrition, University of Illinois, Urbana, IL 61801, USA

## Abstract

**Background:**

The Dietary Reference Intakes (DRI) established acceptable macronutrient distribution ranges (AMDR) for carbohydrates and protein, however little is known about differences in glycemic regulations and metabolic signaling across this range. This study examined metabolic outcomes associated with intake of two diets differing in carbohydrate:protein ratios representing the upper and lower ends of the AMDR.

**Methods:**

Adult, male rats were fed either a high carbohydrate (CHO) diet (60% of energy from carbohydrates, 12% protein, 28% fat; n = 30) or a high protein (PRO) diet (35% carbohydrate, 35% protein, 30% fat; n = 30). Rats were meal-fed 3x/d the respective diets for 10 d and then terminated after overnight food deprivation or 30, 60, 90, 120 min post-prandial (PP). Plasma was collected at each of these points to provide a time course for glucose, insulin and C-peptide. Skeletal muscle and adipose tissues were collected at 0, 30 and 90 min for measurements of basal, early and delayed activation of Akt, p70S6K and Erk 1/2. Data were analyzed by two-way ANOVA.

**Results:**

The CHO group produced a consistently elevated response in plasma glucose, insulin and C-peptide following the meal through the 120 min time course. In addition, Akt and Erk 1/2 activation in adipose was much higher than in skeletal muscle. Conversely, the PRO group PP glucose response was minimal and insulin maintained a response similar to a biphasic pattern. Tissue responses for the PRO group were greater for Akt and p70S6K signaling in skeletal muscle compared with adipose.

**Conclusion:**

Taken together these data suggest that altering CHO:PRO ratios within the AMDR produce different glycemic response patterns accompanied by differential metabolic signaling in skeletal muscle and adipose.

## Background

In the context of the rising incidence of insulin resistance, obesity, and cancer more attention has become focused on improving glycemic control, specifically insulin responsiveness, through altering dietary macronutrient ratios [1,2]. The current Dietary Reference Intakes (DRI) establish a wide range of acceptable macronutrient distributions (AMDR) for carbohydrates, protein, and fat, however very little is known about the effects of these ranges on metabolic regulations and specifically on insulin signaling in skeletal muscle and adipose. However, the wide ranges of potential macronutrient ratios would be expected to produce different insulin responses following a meal [3,4]. Previous studies suggest that decreasing the dietary carbohydrate:protein ratio within the recommended DRI guidelines improves overall glucose homeostasis and insulin responsiveness [1,2], but it is unclear how peripheral metabolic tissues adapt to and mediate this response.

We propose that short-term dietary alterations in carbohydrate:protein ratios fed to phenotypically normal, lean adult rats will produce changes in insulin-mediated intracellular signal pathways - namely the metabolic arm signaling through Akt and p70S6K, and the mitogenic arm signaling through Erk1/2. Investigation into both pathways is supported by previous data in both animal and human studies that have shown altered glycogen metabolism and glucose disposal when fed differing ratios of carbohydrates, protein and fat [5,6], as well as changes in adipogenesis [7]. Furthermore, we suggest that these metabolic changes have a differential response in skeletal muscle and adipose dependent upon carbohydrate load. While both tissues dispose of dietary glucose, they utilize glucose for different metabolic and mitogenic purposes and likely possess different adaptive capabilities.

We developed two isoenergetic test diets with different ratios of carbohydrate:protein representing the DRI guidelines for AMDR [8]. The high carbohydrate:protein ratio diet represents the upper end of the DRI range for carbohydrate intake and the lower end of the protein range and is similar to the typical carbohydrate intake in a Western diet[9]. The reduced carbohydrate:protein diet represents the lower end of the DRI range for carbohydrates and the upper end of the protein range. Previous studies have shown that diets with this composition of reduced carbohydrate:protein ratio enhance glycemic regulations [10]. This study examines the effects of these diets on glycemic regulations including glucose, insulin and C-peptide concentrations and phosphorylation activation of Akt, p70S6K, and Erk1/2 as key regulatory markers of insulin signaling in skeletal muscle and adipose.

## Materials and methods

### Animals and Feeding Protocol

Male Sprague-Dawley rats (274.7 ± 1.5 g; Harlan-Teklad, Indianapolis, IN) were maintained at 24 °C in 12 h light:dark cycle with free access to water. Rats were trained to meal-feed 3x/d consuming either a diet with a high carbohydrate:protein ratio (CHO group) or a diet with a low carbohydrate:protein ratio (PRO group). Treatments were isoenergetic and designed to represent DRI ranges for acceptable macronutrient intakes. Treatments consisted of either 60% of energy from carbohydrate, 12% protein and 28% fat (n = 30; CHO group) or 35% carbohydrate, 35% protein and 30% fat (n = 30; PRO group) (Table 1). The meal pattern consisted of a 4 g breakfast meal (20% of total energy) consumed between 0700-0720, followed by free access to food between 1300 to 1400 (~40% total energy) and 1800 to 1900 (~40% total energy). This feeding pattern was designed to mimic human eating behavior [5]. To assess early metabolic changes, diets were fed for 10 d and rats were terminated by guillotine at 5 time points: prior to the 4 g breakfast meal, and 30, 60, 90 and 120 min following the 4 g breakfast meal (n = 6/time point). Trunk blood was collected at each time point, and liver, gastrocnemius, plantaris, and retroperitoneal fat pads were excised at 0, 30 and 90 min and immediately frozen in liquid nitrogen. Animal protocol was approved by the University of Illinois Institutional Animal Care and Use Committee.

**Table 1:** Diet composition of the CHO and PRO diets

Component	CHO Group		PRO Group	
	*g/kg*	*% energy*	*g/kg*	*% energy*
Whey	128.33	12.0	379.78	35.0
Cornstarch	401.04	37.5	237.36	21.9
Maltodextrin	137.96	12.9	81.65	7.5
Sucrose	102.67	9.6	60.77	5.6
Soybean oil	133.03	28.0	144.48	30.0
Cellulose (fiber)	53.70		3.70	
Mineral mix^1^	37.60		37.60	
Vitamin mix^1^	10.70		10.70	
Choline bitartrate	2.70		2.70	

^1^AIN-93 mineral and vitamin supplements from Harlan-Teklad

### Plasma Analysis

Plasma insulin and C-peptide were measured using commercial RIA kits (Millipore, St. Charles, MO) specific for rat. Plasma glucose was measured using a glucose oxidase/peroxidase method (Invitrogen, Carlsbad, CA).

### Tissue Preparation

Plantaris and adipose were pulverized in liquid nitrogen and homogenized in 2 ml ice cold lysis buffer using a Polytron homogenizer for 30 s. Samples were centrifuged at 10,000 × g for 10 min at 4°C. The supernatant was collected, aliquoted and stored in -80°C until further analysis. Total protein in samples was quantified using bicinchoninic method (Pierce, Rockford, IL). Both plantaris and adipose were analyzed unless otherwise stated. Two rats from each diet (CHO and PRO) and each time point (0, 30, and 90 min) were run on the same gel for phosphorylated protein and a duplicate gel run for total protein.

### Total Akt and Akt Ser473 phosphorylation

Akt analysis was performed using 30 μg whole cell lysates from plantaris and adipose and resolved on 10% SDS-PAGE. Following transfer, nitrocellulose membranes were probed with either rabbit polyclonal Akt antibody or rabbit polyclonal phospho-Akt antibody specific for ser473 (Cell Signaling Technologies, Danvers, MA). The membrane was washed three times with TBS-T then incubated with HRP labeled goat anti-rabbit IgG (Cell Signaling Technologies, Danvers, MA) for 1 h at room temperature. The membrane was again washed three times in TBS-T. Labeling was detected with enhanced chemiluminescence for 1 min and exposed to Kodak Biomax film for 15 s. Quantification of bands was determined via densitometry (Image J; NIH, Bethesda, MD) and activation represented as percent of total protein phosphorylated.

### Total p70S6K

p70S6K analysis was performed using whole cell lysates isolated from plantaris and adipose and resolved on 7.5% SDS-PAGE. Following transfer the membrane was incubated with rabbit polyclonal p70S6K antibody (Bethyl Labs, Montgomery, TX) diluted 1:1500 in Superblock T20 overnight at 4° C with gentle rocking. The membrane was washed three times with TBS-T and incubated with HRP labeled goat anti-rabbit IgG diluted 1:1500 for 1 h at room temperature with gentle rocking. The membrane was again washed three times in TBS-T. Labeling was detected with enhanced chemiluminescence for 1 min and exposed to Kodak Biomax film for 5 s. Quantification of bands was determined via densitometry and activation represented as percent γ and β (phosphorylated) subunit compared with total p70S6K subunits (α+β+γ).

### Total and phospho-Erk 1/2

Whole cell lysate from plantaris and adipose were resolved on 12% SDS-PAGE. Following transfer the membrane was blocked with Superblock T20 for 45 min at room temperature with gentle rocking. The membrane was incubated with either rabbit polyclonal p44/42 MAPK antibody or phospho-p44/42 MAPK antibody specific for thr202 and tyr204 (Cell Signaling, Danvers, MA), both diluted 1:1500 in Superblock T20 overnight at 4 °C with gentle rocking. The membrane was washed three times with TBS-T and incubated with HRP labeled goat anti-rabbit antibody diluted 1:1500 for 1 h at room temperature with gentle rocking. The membrane was again washed three times with TBS-T. Labeling was detected with enhanced chemilumenescence for 1 min and exposed to Kodak Biomax film for 15 s. Quantification of bands was determined via densitometry and activation represented as percent of total protein phosphorylated.

### Statistical Analysis

Values are presented as means ± SEM. Data were analyzed by two-way ANOVA using SPSS Version 15.0 (SPSS Inc., Chicago, IL) with subsequent Tukey post-hoc with diet treatment and time points as independent variables. Statistical significance was set at p < 0.05.

## Results

### Body Weights and Food Intake

Body weights and food intake were not different between diet treatments. Final body weights were 275.7 ± 1.3 g and 273.7 ± 1.6 g in the PRO and CHO treatments, respectively.

### Plasma Glucose, Insulin and C-peptide

Fasting blood glucose in the PRO treatment was 10% higher than the CHO treatment (Table 2) which is consistent with previous studies [3]. Plasma glucose response to the meals differed between diet groups. The CHO treatment exhibited a parabolic shaped curve that increased to a peak at 60 min PP (11.27 ± 0.66 mmol/L) and decreased to near baseline levels by 120 min, whereas the PRO treatment exhibited an early peak in glucose at 30 min PP (9.71 ± 0.20 mmol/L) and was not different from baseline levels at 60 min. Plasma glucose was significantly higher in the CHO group compared to the PRO treatment at 60 min.

**Table 2:** Pre and post-meal plasma insulin, glucose and C-peptide of rats consuming a CHO or PRO diet for 10d^1^

Plasma Measures	Premeal^2^	Postmeal^3 ^30 min	Postmeal^3 ^60 min	Postmeal^3 ^90 min	Postmeal^3 ^120 min
Glucose (mmol/L)†					
PRO	6.61 ± 0.16*	9.71 ± 0.20	7.38 ± 0.25*	8.18 ± 0.33*	8.44 ± 0.51
CHO	6.00 ± 0.12	9.35 ± 0.18	11.29 ± 0.66	9.74 ± 0.52	8.26 ± 0.41
Insulin (pmol/L)					
PRO	99.58 ± 13.65	444.81 ± 80.68	342.33 ± 32.80*	480.82 ± 75.48	396.89 ± 84.70
CHO	93.43 ± 13.66	407.58 ± 92.28	518.35 ± 61.58	544.23 ± 101.69	520.28 ± 103.55
C-peptide (nmol/L)†					
PRO	0.68 ± 0.06	2.52 ± 0.12	2.10 ± 0.10*	2.43 ± 0.15	1.93 ± 0.15
CHO	0.69 ± 0.05	2.59 ± 0.15	2.96 ± 0.08	2.65 ± 0.09	2.38 ± 0.05

^1^Values are ± SEM, n = 6

^2^Following 12 h food deprivation

^3^Samples collected 30, 60, 90 and 120 min following 4 g meal

* < 0.05 between diet

† < 0.05 diet x time interaction

**Figure 1:**
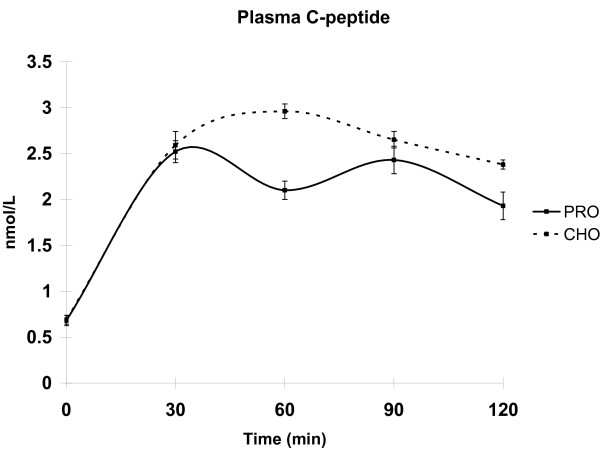
***Plasma C-peptide concentrations *in rats meal-fed CHO or PRO for 10 d**. Samples were taken after 12 h overnight food deprivation and 30, 60, 90, and 120 min PP. Values are means ± SEM. Means with an asterisk are different (p < 0.05) between diets. n = 6/time point.

Consistent with PP plasma glucose concentrations, the insulin and C-peptide curves exhibited different PP response patterns between the diet treatments (Table 2). The PRO treatment produced a PP biphasic insulin response curve with insulin concentrations higher at 30 min PP and 90 min PP, and comparatively decreased at 60 min. PP insulin increased in response to the CHO meal at 30 min PP and remained elevated through the 120 min time course. Diet treatments were significantly different at 60 min. The time-course of insulin release is further characterized by the C-peptide responses (Figure 1).

### Total Akt and Akt Ser473 phosphorylation

**Figure 2:**
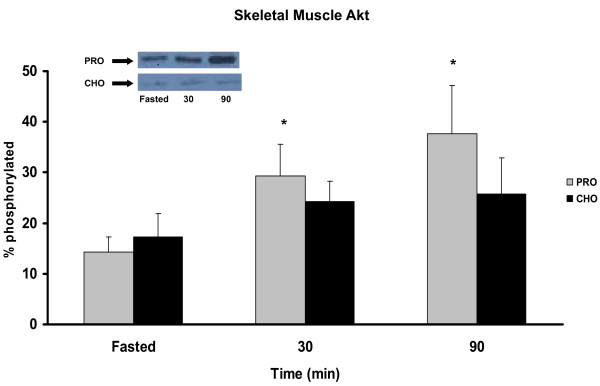
***Akt phosphorylation in skeletal muscle *pre-meal and 30, 90 min PP in rats meal-fed CHO or PRO for 10 d**. Samples were collected after 12 h overnight food deprivation and 30, 90 min PP. Values are means ± SEM. Means with an asterisk are different compared to time 0 (p < 0.05), n = 6/timepoint.

**Figure 3:**
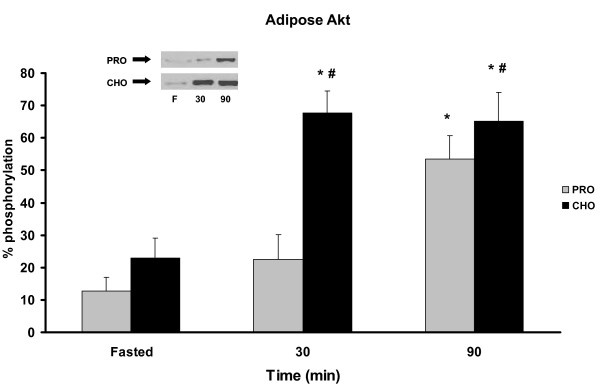
***Akt phosphorylation in adipose *pre-meal and 30, 90 min PP in rats meal-fed CHO or PRO for 10 d**. Samples were collected after 12 h overnight food deprivation and 30, 90 min PP. Values are means ± SEM. Means with an asterisk are different compared to time 0 (p < 0.05), and hash mark is different between diets (p < 0.05), n = 6/timepoint.

Intracellular Akt levels exhibited differential expression in skeletal muscle and adipose depending on the diet treatment. Animals consuming the PRO diet displayed a 2- to 3-fold increase in Akt activation in skeletal muscle at 30 and 90 min PP (p < 0.05) while the CHO was not significantly different from baseline (Figure 2). In adipose tissue, the CHO group exhibited significant elevation of phosphorylated Akt in adipose in response to the meal at 30 min PP and remained elevated at 90 min (Figure 3). Animals in the PRO group had delayed activation of Akt elevated only at 90 min PP.

### Total p70S6K

**Figure 4:**
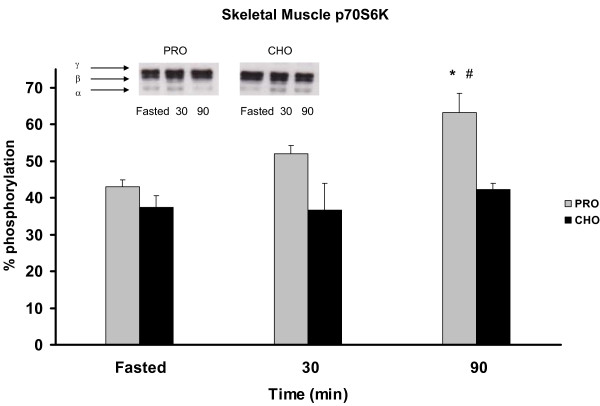
***p70S6K phosphorylation in skeletal muscle *in rats meal-fed CHO or PRO for 10 d**. Samples were collected after 12 h overnight food deprivation and 30, 90 min PP. Values are means ± SEM. Means with an asterisk are different compared to time 0 (p < 0.05). Means with a hash mark are different between diets (p < 0.05). n = 6/time point.

Meal responses for p70S6K in skeletal muscle were different between treatment groups and similar to Akt responses. The PRO group increased p70S6K activation at 30 min PP with continued increases at 90 min PP (Figure 4). Animals consuming the CHO diet failed to stimulate p70S6K activity in skeletal muscle in response to the meal. The PRO treatment exhibited 12% and 20% greater activation of the γ (hyperphosphorylated) subunit at 30 and 90 min (p < 0.05) PP respectively compared with the CHO treatment. P70S6K in adipose tissue was only faintly detected in either diet treatment likely due to insufficient protein extracted during processing (data not shown).

### Total Erk and Erk Thr202/Tyr204 (p42/44 MAPK) phosphorylation

**Figure 5:**
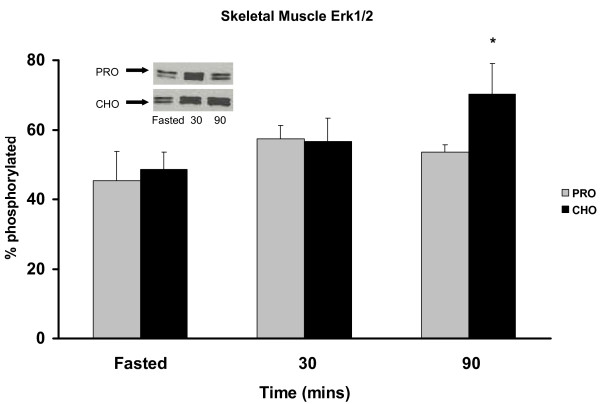
***Erk 1/2 phosphorylation in skeletal muscle *pre-meal and 30, 90 min PP in rats assigned to CHO or PRO diet after 10 d of meal-feeding**. Samples were collected after a 12 h overnight food deprivation and 30, 90 min PP. Values are means ± SEM. Means with an asterisk are different from time 0 (p < 0.05). n = 6/time point.

**Figure 6:**
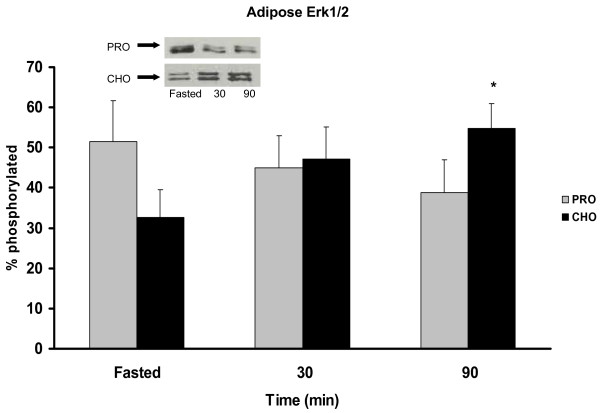
***Erk 1/2 phosphorylation in adipose *pre-meal and 30, 90 min PP in rats assigned to CHO or PRO diet after 10 d of meal-feeding**. Samples were collected after a 12 h overnight food deprivation and 30, 90 min PP. Values are means ± SEM. Means with an asterisk are different from time 0 (p < 0.05). n = 6/time point.

The CHO group exhibited a 22% increase in Erk1/2 phosphorylation at 90 min PP in skeletal muscle (Figure 5). This same trend was found in the adipose for the CHO group (Figure 6) with a 23% increase in phosphorylation at 90 min PP. Conversely, the PRO group had no significant response to the meal in either skeletal muscle or adipose.

## Discussion

DRI guidelines establish wide ranges of acceptable macronutrient intakes implying that individuals can select healthy intakes at any point within these ranges. However, little is known about metabolic responses, particularly insulin-mediated signaling, at the upper and lower ends of these ranges. The current study demonstrates significant differences in insulin signaling with carbohydrate and protein intakes at the higher and lower ends of the DRI range.

Animals in both the PRO and CHO treatment groups exhibited PP glycemic responses, however the responses differed between treatments. These glycemic regulations are measured at the systemic level by insulin, glucose and C-peptide, and at the tissue level by Akt, p70S6K, and Erk1/2. Previous studies have shown diets with reduced carbohydrates (<150 g/d) and increased protein (>1.5 g/kg) improve glycemic regulations in normal [11], obese [12,13] and diabetic [14-18] subjects. This study using phenotypically normal rats also found improved glycemic regultions including reduced PP plasma glucose and enhanced biphasic-like insulin and C-peptide response. The PRO group also had higher fasting blood glucose previously shown to be due to increased hepatic gluconeogenesis and reduced reliance on glycogen [19]. These differences in PP glycemic responses to the diets are expected due to differences in the dietary carbohydrate load, however the increased PP glucose response with the higher carbohydrate diet indicates a shift in focus of insulin signaling from skeletal muscle to adipose tissue to maintain glucose homeostasis [10].

The differences in glycemic regulation are particularly evident during the early period of the insulin response (30 min PP). Classically, the early phase response of plasma insulin corresponds to release of endogenous insulin stores from the pancreas, which is believed to occur in the first 10-20 min PP [20]. However, this classical view is based upon acute-phase glucose stimulated cultured beta cells, and direct glucose infusions into animals and human subjects. When consuming a mixed meal, the bi-phasic response exhibits a lag due to the mechanical digestion required to release the glucose load into systemic circulation. Therefore, it is legitimate to believe the responses observed in this study at 30 min PP are indicative of an early phase response. The late phase, reflecting insulin concentrations between 60 min PP, represents newly synthesized insulin in response to circulating glucose concentration from a meal [21]. Insulin responses are often accompanied by C-peptide measures as insulin and C-peptide are released from the beta cell in a 1:1 molar ratio. However, C-peptide has a half-life of 30 min and little biological activity once released. Thus, it serves to corroborate insulin measurements which can often be skewed by its relatively short half-life of ~5 min and high metabolic activity. Findings in the present study show similar response patterns between insulin and C-peptide. These findings suggest Phase I insulin response is largely insensitive to the absolute amount of carbohydrates in the meal, while the second phase appears to be more adaptive. Our data supports this because PP plasma insulin responses in the first 30 min were not different between the CHO and PRO groups (+331 pmol/L vs. +345 pmol/L, respectively) even though the CHO meal contained 70% more glucose (2.4 g vs. 1.4 g). Comparing these changes in plasma insulin with the amount of carbohydrate in the meal [i.e. insulin concentration (pmol/L) divided by grams of carbohydrate in the meal], the CHO group had a Phase I insulin response of 130 pmol/L per grams of dietary glucose at 30 min PP while the response in the PRO group was 246 pmol/L per gram of glucose. This blunted response in the CHO group resulted in elevated plasma glucose at 60 min PP and a prolonged hyperinsulinemia at 120 min. The prolonged insulin response in the CHO group is consistent with observations in human studies for subjects fed high carbohydrate diets with similar compositions [5,13].

In addition to different glycemic regulations, altered CHO:PRO ratios created signaling differences in energy sensing metabolic pathways in skeletal muscle and adipose tissues. Akt is an insulin-stimulated upstream signal kinase which triggers release of GLUT4 for glucose transport and downstream activation of protein synthesis via mTOR-mediated p70S6K activation [22]. Surprisingly, the PRO diet produced a greater Akt response in skeletal muscle than the CHO diet which generated higher PP insulin release. Hence the PRO diet produced a greater Akt response with lower plasma insulin reflecting greater insulin sensitivity in skeletal muscle. This difference was further evident downstream at p70S6K which is sensitive to the insulin signal via Akt and also to amino acid availability via mTOR activation by intracellular leucine [23]. The combination of reduced carbohydrates and increased protein in the PRO diet produced the greatest sensitivity in skeletal muscle Akt and p70S6K signaling. These findings are in agreement with previous reports that glucose uptake into skeletal muscle [3] and muscle protein synthesis [24] are higher in PRO vs. CHO diets and consistent with the dysregulation of insulin signaling observed with insulin resistance.

Contrary to the findings in skeletal muscle, the Akt response in adipose was higher for the animals consuming the CHO diet. At 30 min PP Akt in the CHO group was approximately 3-fold higher than the PRO group. Again, this is consistent with report of greater glucose uptake in animals adapted to a CHO diet [3] and suggests that animals chronically consuming a CHO diet will preferentially rely on adipose to dispose of excess glucose after a high carbohydrate meal.

Furthermore, Erk1/2 levels associated with the CHO diet are increased in adipose. Erk1/2, when elevated, is characteristically a marker of cell growth and division, and recently linked to increases in adipocity [7]. Thus, when taken together, the meal responses of increased Akt levels and increased glucose uptake appear to be shunting glucose disposal into the adipose which may stimulate adiposity via activation of the Erk1/2 pathway. Contrary to the metabolic pattern of the CHO diet, the PRO diet decreases Erk1/2 levels in adipose and instead increases p70S6k levels in skeletal muscle consistent with higher protein turnover and greater energy expenditure [25].

## Conclusions

This study demonstrates that while normal animals have the ability to maintain glucose homeostasis across the full range of the DRI AMDR, chronic consumption of a diet with altered CHO:PRO ratios produce different glycemic regulations resulting in different signaling responses in skeletal muscle and adipose. Animals chronically consuming the CHO diet produced greater metabolic signaling in adipose tissue to handle excess glucose and blunted signaling in skeletal muscle consistent with interpretation of insulin resistance. Conversely, animals consuming the PRO diet produced greater metabolic signaling in skeletal muscle with little signaling in adipose. While these data suggest that consuming an increased CHO:PRO diet may have detrimental effects on insulin sensitivity, the long-term significance of these metabolic differences warrants further investigation.

## Competing interests

Supported by grants from the National Dairy Council/DMI and the Illinois Department of Agriculture (CFAR).

Author Disclosures: SD has no conflict of interest; DKL contributes to the NDC and National Cattleman's Beef Association speaker's bureaus, and a nutrition consultant to the Egg Nutrition Center.

## Authors' contributions

SD designed study and carried out all experimental procedures and analyses, and drafted the manuscript. DKL helped conceive and design study, edited the manuscript, and approved final submission. All authors have read and approved the final manuscript.

## Abbreviations used

AMDR: Acceptable Macronutrient Distribution Range; CHO: increased carbohydrate:protein ratio diet; DRI: Dietary Reference Intakes; Erk1/2: extracellular signal related kinase 1/2; HRP: horseradish peroxidase; MAPK: mitogen activated protein kinase; mTOR: mammalian target of rapamycin; p70S6K: 70-kDa ribosomal protein S6 kinase; PRO: decreased carbohydrate:protein ratio diet; PP: post-prandial; TBS-T: tris buffered saline with tween.
